# Fe^3+^ and Al^3+^ removal by phosphate and hydroxide precipitation from synthetic NMC Li-ion battery leach solution

**DOI:** 10.1038/s41598-023-48247-6

**Published:** 2023-12-05

**Authors:** Alexander Chernyaev, Jianxin Zhang, Sipi Seisko, Marjatta Louhi-Kultanen, Mari Lundström

**Affiliations:** Department of Chemical and Metallurgical Engineering, School of Chemical Engineering, Aalto University, 00076 Aalto, Finland

**Keywords:** Chemical engineering, Process chemistry

## Abstract

The removal of trivalent iron and aluminum was studied from synthetic Li-ion battery leach solution by phosphate and hydroxide precipitation (pH 2.5–4.25, *t* = 3 h, *T* = 60 °C). Phosphate precipitation exhibited both crystal nucleation initiation (pH 2 vs. pH 3) as well as complete (~ 99%) Fe and Al removal at lower pH compared to hydroxide precipitation (pH 3 vs. 3.5). The precipitation time of phosphate was shorter (40 min) than that of hydroxide precipitation (80 min). At pH 4 the loss of valuable metals (Li, Ni, Co) in the precipitate was negligible in the phosphate cake, whereas in the hydroxide process the co-precipitation was 4–5% for Li, Ni and Co. The filtration rate of phosphate precipitate was shown to be significantly faster. The presence of fluoride did not have any notable effect on phosphate precipitation, whereas in hydroxide precipitation, it potentially had a negative effect on aluminum extraction.

## Introduction

Lithium-ion batteries (LIBs) can contain various elements such as Co, Cu, Ni, Li, Mn, Al, Fe, F, and P. The cathode is of the highest value^1^, with a common compound of LiCoO_2_ (LCO), LiNi_x_Mn_y_Co_z_O_2_ (NMC), LiFePO_4_ (LFP), LiMn_2_O_4_ (LMO), and LiNi_0.8_Co_0.15_Al_0.05_O_2_ (NCA)^1–3^. In state-of-the-art battery waste, the most valuable elements are considered to be Ni, Co, and Cu. Further, elements such as Li^4^, Mn^5,6^, and graphite^7–9^ have aroused increasing interest for recycling even on industrial scale. However, a few elements such as F, Fe, and Al are considered impurities in LIB recycling processes since their recycling is regarded as unprofitable and can affect the recovery of the high-value elements in the process.

In the recycling process, battery cells are crushed and separated (e.g., by screening), resulting in a small particle size fraction concentrated with battery metals, referred to as black mass^10^. In NMC-based black mass, the Ni content has been reported to be 2–16%, that of Mn 7–24%, Co 2–7.5%, Cu 2.2–15%, Fe 0–1%, and Al 0.6–12.3%^11–13^. F^–^ is an impurity element that typically originates from LiPF_6_, which is the most commonly used electrolyte salt in the current LIB market^12,14^. The extraction and recovery of valuable metals from black mass have been extensively studied^15–17^. Only now the focus of research has been shifting towards investigating the role of low-value impurities, such as Fe, Al, and F in battery recycling.

Black mass can be refined via pyro-hydrometallurgical or hydrometallurgical processing routes into battery-grade purity chemicals, including graphite^1,18–21^. For instance, a novel method for pyro-hydrometallurgical method of black mass recycling was reported by González et al.^21^, where black mass underwent carbochlorination process followed by water leaching. In hydrometallurgical treatment, the black mass undergoes leaching in inorganic acid, typically H_2_SO_4_ or HCl^18,22^. To complete the leaching, the active material metal oxides are reduced with a reducing agent such as H_2_O_2_ or even with metallic elements such as Cu or Fe^23,24^. Not only valuable metals but also impurities (Fe and Al) dissolve into the pregnant leach solution (PLS) and need to be removed^11,25^ before further separation and recovery of battery-grade salts such as MnSO_4_, CoSO_4_, NiSO_4_, and Li_2_CO_3_. Alternatively, dissolved copper can be recovered prior to Fe-Al removal.

It has been suggested that mechanical separation and recovery of Al—prior to metallurgical treatment of black mass—would have a substantial positive impact on the carbon footprint of the overall recycling process^26^. However, the technological readiness level of mechanical Al separation varies, and some aluminum is commonly found in black mass. In the literature on the state of the art, for instance, the concentration of Al in LIB PLS is most often < 1 g/L, although concentrations of > 2 g/L have also been reported^22,24,27–31^. Hydroxide precipitation has been widely used in solution purification to remove Fe and Al on laboratory scale. However, it is known that the precipitation of aluminum as hydroxide causes co-precipitation, i.e., losses of valuable battery metals such as Ni, Li, and Co in the precipitate^11,27,32–34^. Moreover, the poor filterability of Al(OH)_3_ solids poses a significant challenge in processing^11,27^. Liu et al.^31^ precipitated Fe^3+^ from sulfuric acid solution using NaHCO_3_ solution in pH adjustment and phytic acid (C_6_H_18_O_24_P_6_) to precipitate Al^3+^ at pH 3.5. In their study it was found that phytic acid caused only minimal Ni and Co co-precipitation in Al-phytate cake. As a result, Al-phytates ($Al_{x} C_{6} H_{18 - 3x} P_{6} O_{24} \cdot nH_{2} O$) were precipitated, and treated at 1000 °C to form Al(PO_3_)_3_^31^. An alternative strategy for aluminum removal was proposed by Fang et al.^35^, where aluminum was first dissolved from black mass in excess NaOH and recovered as sodium meta-aluminate, followed by precipitation as pure aluminum hydroxide (99%), with the pH value adjusted to 6 by H_2_SO_4_, yielding Al(OH)_3_ and Na_2_SO_4_ filtrate. This method allowed selective recovery of Ni, Co, Mn, and Li in the following step, as the active material did not dissolve in NaOH and was filtered and leached separately in H_2_SO_4_ media.

Highly soluble phosphates (e.g., sodium phosphate or phosphoric acid) have been suggested as potentially effective alternative precipitation agents to highly soluble hydroxides. According to Gu et al.^36^, phosphate and hydroxide precipitation can occur in a sulfate system. The use of H_3_PO_4_ to recover FePO_4_ and AlPO_4_ was recently patented by Northvolt AB^37^, one of the largest Li-ion battery manufacturers in Europe. Masambi et al.^38^ investigated iron phosphate precipitation from chloride solutions containing iron (45 g/L), copper (3 g/L), and nickel (3 g/L) at *pH* = 1–3 (*T* = 40–90 °C). Iron removal of 98.8% was achieved with minor co-precipitation of nickel (0.5%) and copper (2.8%) at *T* = 40 °C and *pH* = 1. When the temperature was raised from 40 to 60 °C, iron removal was increased to 99.9%, but nickel and copper losses increased to 9.2% and 33.1%, respectively. A further increase in pH promoted the co-precipitation of nickel and copper. Additionally, Zhang et al.^27^ demonstrated that 99.9% of Al could be recovered by phosphate precipitation with only 2.34% co-precipitation of other metals (Ni, Co, Mn, Li) at pH 3.5 (1.1 times theoretical Na_3_PO_4_ dosage, 80 °C, 30 min). Complete precipitation of aluminum and fluoride in LiFePO_4_ battery leach solution was also reported by Jie et al.^30^ (pH = 3.9, 25 °C). The main products reported by Jie et al., were FePO_4_, Fe(OH)_3_, AlPO_4_, and FeF_3_, in a solution where the concentration of iron was more than 10 times higher than that of aluminum. Besides, Klaehn et al.^39^ in their work investigated removal of Fe and Al at 45 °C from the LIB leach solution using (NH_4_)_2_HPO_4_ for phosphate and NH_4_OH for hydroxide processes. The phosphate process was found to be more efficient in terms of Fe and Al removal, and lower co-precipitation of Co and Ni was observed.

The possible chemical reactions of Fe and Al in phosphate and hydroxide precipitation in the presence of F^–^ are presented in Table 1. The iron in divalent form precipitates as hydroxide at neutral pH (Eq. 1), while the metals in trivalent form (Fe^3+^, Al^3+^) precipitate as hydroxides at pH 3–5 (Eq. 2 and Eq. 3). Additionally, they readily precipitate as phosphates at low pH values (3–4) (Eq. 4 and Eq. 5). Furthermore, phosphate can rapidly and stepwise dissociate into H^+^ and PO_4_^3–^ (Eqs. 6–8). In acidic conditions, fluoride is known to form highly corrosive and toxic HF acid (Eq. 9). The stability constant of the reactions of fluoride with aluminum (Eqs. 10–14) and iron (Eqs. 18 and 19) are higher than with H^+^ (Eq. 9), hence fluoride can be preferentially complexed with the trivalent metals in question. Additionally, the presence of aluminum may have a positive effect on the recycling process as the formation of HF could be minimized, and consequently, the fluoride could be removed from the solution together with iron and aluminum (Eqs. 15 and 17).

**Table 1:** Stability constants for the formation of solids from iron and aluminum at 60 °C (HSC 10 Chemistry, software version 10.0.5.16, Metso Outotec).

Reaction	Log K (60 °C)	Equations
$$Fe^{2 + } + OH^{ - } = Fe\left( {OH} \right)_{2}$$(s)	16	(1)
$$Fe^{3 + } + OH^{ - } = Fe\left( {OH} \right)_{3}$$(s)	36	(2)
$$Al^{3 + } + OH^{ - } = Al\left( {OH} \right)_{3}$$(s)	31	(3)
$$Fe^{3 + } + PO_{4}^{3 - } = FePO_{4}$$(s)	27	(4)
$$Al^{3 + } + PO_{4}^{3 - } = AlPO_{4}$$(s)	19	(5)
$H^{ + } + H_{2} PO_{4}^{ - } = H_{3} PO_{4}$	2.5	(6)
$H^{ + } + HPO_{4}^{2 - } = H_{2} PO_{4}^{ - }$	7	(7)
$H^{ + } + PO_{4}^{3 - } = H_{2} PO_{4}^{2 - }$	12	(8)
$H^{ + } + F^{ - } = HF$	3	(9)
$Al^{3 + } + F^{ - } = AlF^{2 + }$	7	(10)
$Al^{3 + } + 3F^{ - } = AlF_{3}$	17	(11)
$Al^{3 + } + 4F^{ - } = AlF_{4}^{ - }$	18	(12)
$Al^{3 + } + 5F^{ - } = AlF_{5}^{2 - }$	20	(13)
$Al^{3 + } + 6F^{ - } = AlF_{6}^{3 - }$	20	(14)
$Al^{3 + } + 2F^{ - } + H_{2} O = AlF_{2} OH\left( s \right) + H^{ + }$	6^a^	(15)
$Al^{3 + } + 3F^{ - } + H_{2} O = AlF_{3} OH^{ - } + H^{ + }$	10^a^	(16)
$$Fe^{3 + } + 3F^{ - } = FeF_{3}$$(s)	12	(17)
$Fe^{3 + } + 2F^{ - } = FeF_{2}^{ + }$	9	(18)
$Fe^{3 + } + F^{ - } = FeF_{{}}^{2 + }$	13	(19)

^a^At 25 °C, Ntuk et al.^50^.

In this work, phosphate and hydroxide precipitation was investigated comprehensively. The investigated outputs include precipitation time, iron and aluminum precipitation efficiencies, undesired co-precipitation of valuable metals such as Ni, Co, co-precipitation, with novel results on Li behavior in these purification processes. Also, the novelty of this research includes the use of phosphoric acid as an aqueous source of phosphate ions, real-time tracking of particle formation, pH as well as temperature. Additionally, the novelty aspects include integrated precipitation and filtration steps. Obtained filtration parameter values of specific cake resistance and compressibility can be used further when sizing and designing process scale filters. The results gained in this work can pave the way for more efficient removal of iron and especially aluminum using phosphoric acid in the solution purification stage of LIB recycling.

## Materials and methods

### Reagents and synthetic pregnant leach solution

Synthetic PLS was prepared to mimic real PLS from the leaching of NMC-rich battery waste^23,24,40^, as shown in Table 2. The reagents used in synthetic PLS preparation were Li_2_SO_4_·H_2_O (Sigma Life Science, ≥ 99%), NiSO_4_·6H_2_O (Alfa Aesar, 98%), MnSO_4_·H_2_O (VWR chemicals, ACS/Reag. Ph.Eur.), CoSO_4_·7H_2_O (Alfa Aesar, ≥ 99%), Al_2_(SO_4_)_3_·14–18H_2_O (Alfa Aesar, 97 + %), CuSO_4_·5H_2_O (Sigma-Aldrich, ≥ 98%), Fe_2_(SO_4_)_3_· nH_2_O (VWR chemicals, GPR Rectapur), and LiPF_6_ (98%, Thermo Scientific). Sulfuric acid (H_2_SO_4_, 95–97%, VWR Chemicals) was used to prepare the leach solution with a target concentration of 0.1 M H_2_SO_4_. LiOH (powder, ≥ 98%) was dissolved in water to prepare a 2.5 M solution used for increasing and adjusting the pH of the PLS. Phosphoric acid (H_3_PO_4_, 85%, Alfa Aesar) was used in phosphate precipitation. In composition, it is assumed that the solution mimics a PLS where copper removal has been already conducted before Fe–Al precipitation^34,41^; therefore only traces of dissolved copper (approx. 100 ppm) were added in the initial synthetic PLS^11^. The added fluoride concentration (0.7 g/L) is in line with black mass leach solutions obtained by Porvali et al.^42^.

**Table 2:** Synthetic PLS composition (g/L).

Li^+^	Ni^2+^	Mn^2+^	Co^2+^	Al^3+^	Cu^2+^	Fe^3+^	P^a^	F^–b^
4	11	11	5	4.3	0.1	1.7	0.306	0.7

^a^Applies to phosphate test series (P1–P5, P11FL, PF12, and PF14).

^b^Applies to fluoride test series (PF14 and PF15).

### Precipitation test procedure

The experimental series included phosphate precipitation (P1–P5) and hydroxide precipitation (P6–P9), as well as studies of the impact of F^–^ in both phosphate and hydroxide systems (P12–P15), as shown in Table 3. Precipitation tests (P1–P11FL) were conducted with particle growth tracking in a 100 mL reactor flask in a jacketed system (Easymax 402). The initial solution (PLS) volume was 60 mL, it was pre-heated to 60 °C, and agitation rate was kept constant at 300 rpm. The pre-selected target pH values were in the range of 2.5–4.25, i.e., targeting Fe^3+^ and Al^3+^ precipitation^11,33,43^. The pH was adjusted using 2.5 M LiOH solution in both the phosphate and hydroxide precipitation series while avoiding introducing Na^+^ to the solution from the commonly used neutralization agent NaOH^11^. LiOH was added to the solution using a peristaltic pump (Masterflex L/S) at a constant rate of 0.5 mL/min to reach the target pH value (2.5, 3, 3.5, 4, 4.25) and the pH was measured online throughout the test. Once the target pH was attained, the experiment was initiated and continued for 3 h. The target pH value was maintained by adjusting the pH with 2.5 M LiOH or 1 M H_2_SO_4_. Before increasing the pH in tests P1–P5, P11FL, PF12 and PF14 (Table 3), 0.65 ml of H_3_PO_4_ (85%) was added to the solution. Additionally, focused beam reflectance measurement (FBRM, Particle Track G400) was employed in all tests to track the precipitation progress by measuring the count rate of particle chord length distribution with a data recording interval of 2 s. By combining FBRM and pH measurement, the precipitation behavior as a function of pH could be investigated.

**Table 3:** Fe-Al precipitation series from synthetic LIB PLS (H_2_SO_4_ = 0.1 M, T = 60 °C, t = 180 min).

Exp.	pH	Precipitate	
		Hydroxide	Phosphate
P1	2.5		✓
P2	3		✓
P3	3.5		✓
P4	4		✓
P5	4.25		✓
P6	3	✓	
P7	3.5	✓	
P8	4	✓	
P9	4.25	✓	
P10FL^a^	4	✓	
P11FL^a^	4		✓
PF12	3.5		✓
PF13	3.5	✓	
PF14^b^	3.5		✓
PF15^b^	3.5	✓	

^a^Filterability tests.

^b^Fluoride containing solution.

The tests with fluoride present (PF12–PF15) in the solution were carried out using a different experimental setup, which consisted of a glass beaker (200 mL), magnet stirring bar, magnetic hotplate stirrer (IKA RT 10) employing 350 rpm agitation, a pH electrode (InLab Expert Pro, Mettler Toledo), and a burette. The H_3_PO_4_ was added to the hot solution (60 °C) before increasing the pH. LiOH was added dropwise, and the pH was measured. After the target pH (3.5) was reached, the time counting was initiated. The pH was adjusted every 30 min. Since all experimental work was carried out under a fume hood, a gas detector (GFG micro III) was used to detect HF.

Once the experiments (excl. P10FL and P11FL) were complete, the slurry was vacuum filtered with a Buchner funnel. After slurry filtration was finished and the solution had been collected, the cake was washed with acidified water at the corresponding test pH to avoid precipitation of PLS metals in the cake. The final volume of the solution and wash water was measured, and solution samples were diluted with 0.2 M HNO_3_. The filter paper used was Whatman grade 50 (~ 2.7 μm) for phosphate precipitate separation and grade 54 (~ 22 μm) for hydroxide cake. The cakes were dried in an oven for 20 h at 60 °C before their total weight was measured and a sample of each cake (approx. 200 mg) was transferred to a separate 50 mL volumetric flask and digested in 0.5 M HCl at 80 °C. The cooled solutions were analyzed by flame atomic absorption spectroscopy (AAS, Varian AA240). The aluminum and phosphorus content was analyzed with an inductively coupled plasma optical emission spectrometer (ICP-OES, Perkin Elmer, Optima 7100 DV).

The co-precipitated metal content (Ni, Co, Mn, Li) in the cake was calculated using Eq. (20):

20 $$E\left( \% \right) = \left( {\frac{{c_{cake}^{Me} \times m_{tot}^{cake} }}{{c_{i}^{Me} \times V_{i}^{sol} }}} \right) \times 100$$

where $c_{cake}^{Me}$ is the metal concentration in the cake (g/kg), $m_{tot}^{cake}$ is the total mass of the cake (kg), $c_{i}^{Me}$ is the initial metal concentration in the leach solution (g/L), and $V_{i}^{sol}$ is the volume of the starting leach solution (L). The iron and aluminum extraction was calculated using Eq. (21):

21 $$E\left( \% \right) = 100 - \left( {\frac{{(c_{f} \times V_{f} ) + \left( {c_{ww} \times V_{ww} } \right)}}{{\left( {c_{i}^{Me} \times V_{i}^{sol} } \right)}} \times 100} \right)$$

where $c_{f}$ is the concentration of metal in the final solution (g/L), $V_{f}$ is the final volume of the solution (L), $c_{ww}$ is the concentration of metal in wash water (g/L), and $V_{ww}$ is the final volume of wash water (L). The initial and final concentration of metals are in Table S1. It should be noted that addition of LiOH causes also increase of Li-ions concentration in the solution, which has been taken into account in recovery calculations.

Viscosity measurement was carried out using a Brookfield DV-E viscometer at (22 °C). The particle size distribution (PSD) of the precipitates was analyzed with a laser diffraction particle size analyzer (Master Sizer 2000, Malvern Panalytical). The dry cakes were crushed in a mortar and analyzed using XRD (Malvern Panalytical X’Pert) with Cu K_α_ radiation (wavelength 1.5405 Å) at 40 kV and 45 mA, using a step size of 0.013°. The qualitative elemental analysis of the precipitates was analyzed by scanning electron microscope (SEM, MIRA 3, Tescan, Czech Republic) equipped with an UltraDry Silicon Drift energy-dispersive X-ray spectrometer (EDS) and NSS microanalysis software (Thermo Fisher Scientific, USA).

### Filterability tests

**Figure 1:**
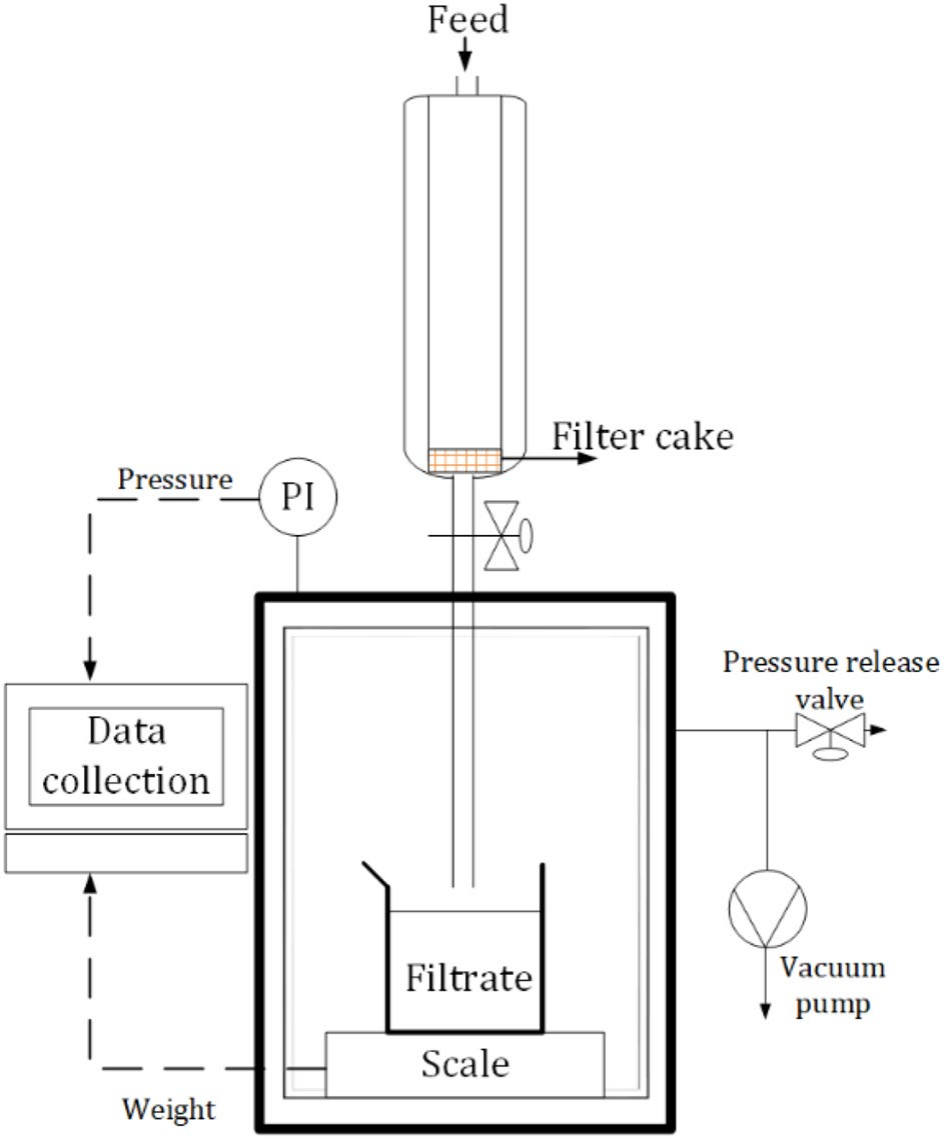
Schematic diagram of vacuum filtration test setup.

The filtration experiments, P10FL and P11FL (Table 3), were carried out with a lab-scale vacuum filtration unit (Fig. 1). Data collection was performed every 2 s with an accuracy of 0.01 bar for pressure and 0.01 g for mass. The filter paper used was Whatman grade 50 (2.7 μm) with a 50 mm diameter. The constant vacuum filtration experiments were conducted at various pressure differences over the cake (400, 600, and 800 mbar).

The filtration rate data (Fig. 5b) was plotted as time/volume (s/m^3^) vs. volume (m^3^) as shown in Fig. S2, and the slope of the linear segment was acquired and used to calculate the specific average cake resistance ($\alpha_{av}$, m/kg) using Eq. (22)^44^:

22 $$slope = \frac{{\alpha_{av} c\mu_{l} }}{{2A^{2} \Delta P}}$$

where $c$ is the feed slurry concentration (kg/m^3^), ${\mu }_{l}$ is viscosity (Pa s), $A$ is the filter area (m^2^), and $\Delta P$ is the pressure difference (Pa). Cake compressibility, $n$, was calculated as the slope of pressure and average cake resistance, both converted to logarithmic values (Eq. 23)^45^:

23 $$\alpha_{av} = \alpha_{0} \Delta P^{n}$$

where ${\alpha }_{0}$ is the specific cake resistance per unit of applied pressure (m/kg kPa^–n^). The experimental values used to calculate the compressibility index are outlined in Fig. S2, Table S2, and Table S3.

## Results

### Fe and Al precipitation

**Figure 2:**
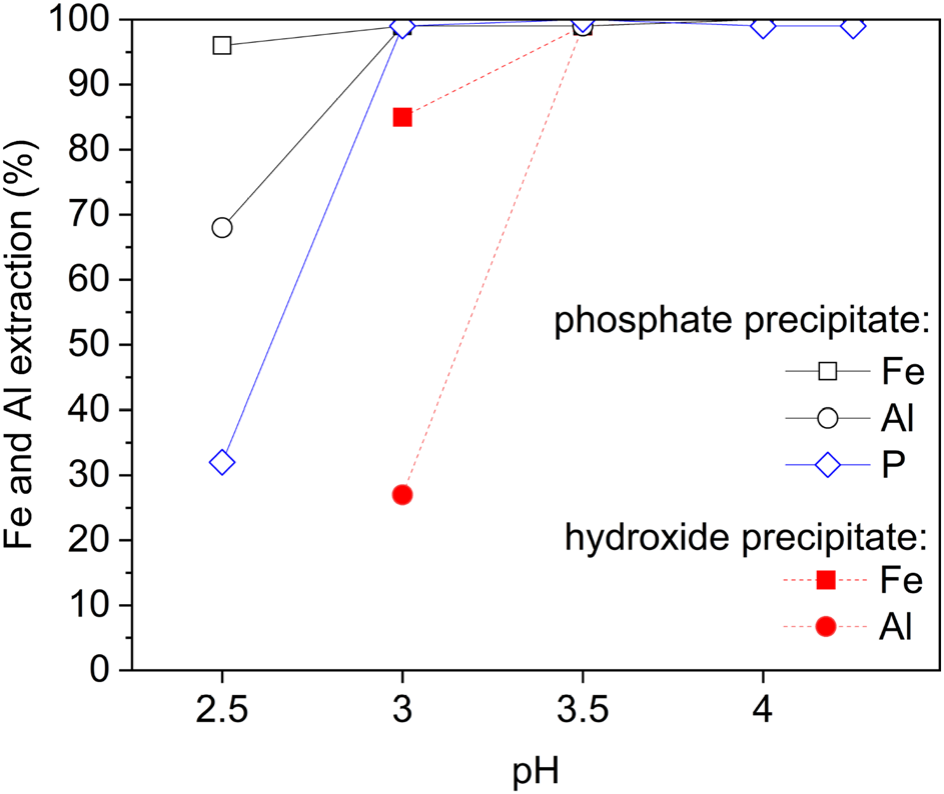
Extraction of iron, aluminum, and phosphorus in phosphate (empty symbols) and hydroxide (filled symbols) precipitation experiments P1–P9 (t = 180 min, T = 60 °C, ω = 300 rpm).

When comparing the simultaneous precipitation of iron and aluminum, phosphate precipitation was shown to perform better at a lower pH (pH 3, 99% recovery) than hydroxide precipitation (pH 3.5, ~ 99% recovery). These results are in line with the thermodynamic modeling by Gu et al.^36^. In this work, only ~ 5 mg/L of Fe and Al remained in the solution during phosphate precipitation (Table 4). In hydroxide precipitation, 0.2 mg/L of Fe and 5.6 mg/L of Al remained in the solution, which is in agreement with the literature^30^. In both studied systems, iron was shown to precipitate at a lower pH when compared to aluminum (Fig. 2 and Table 4). In the current work, soluble iron was present in trivalent form (Fe^3+^), as any dissolved divalent iron in PLS would be oxidized by the active materials^23^. Besides, divalent iron removal from the PLS is known to occur at higher pH (Eq. 4), and Fe(OH)_2_ precipitates typically at pH ~ 6–10^46,47^.

**Table 4:** Concentration of remaining iron, aluminum, and phosphorus in the filtrate of experiments P1–P9 (t = 180 min, 60 °C, ω = 300 rpm).

pH	Phosphate process			Hydroxide process	
	Fe (mg/L)	Al (mg/L)	P (mg/L)	Fe (mg/L)	Al (mg/L)
2.5	57.7	1615.0	1305	–	–
3	6.0	4.0	17.3	288.0	3121.0
3.5	0.2	1.3	7.3	0.2	5.6
4	0.0	3.0	19.0	0.3	6.0
4.25	0.2	1.3	18.8	0.0	0.4

Complete (100%) precipitation of Fe (filtrate with < 1 mg/L), and particularly of Al (filtrate with < 2 mg/L) could be achieved at a lower pH (3.5) in the phosphate precipitation process than with the hydroxide precipitation process (4.25), as shown in Table 4. Additionally, the consumption of LiOH was 2–5% lower in the phosphate process compared to hydroxide at pH 3.5–4.25 (Fig. S1). Phosphorus was precipitated at pH 3 and remained on the level of 10–20 mg/L in all test filtrates (P1–P5). The phosphorus was found in precipitates according to the elemental maps obtained by EDS (Figs. S5–S8). Besides, sulfur was found in both precipitates, which was higher in precipitates obtained in hydroxide system.

**Figure 3:**
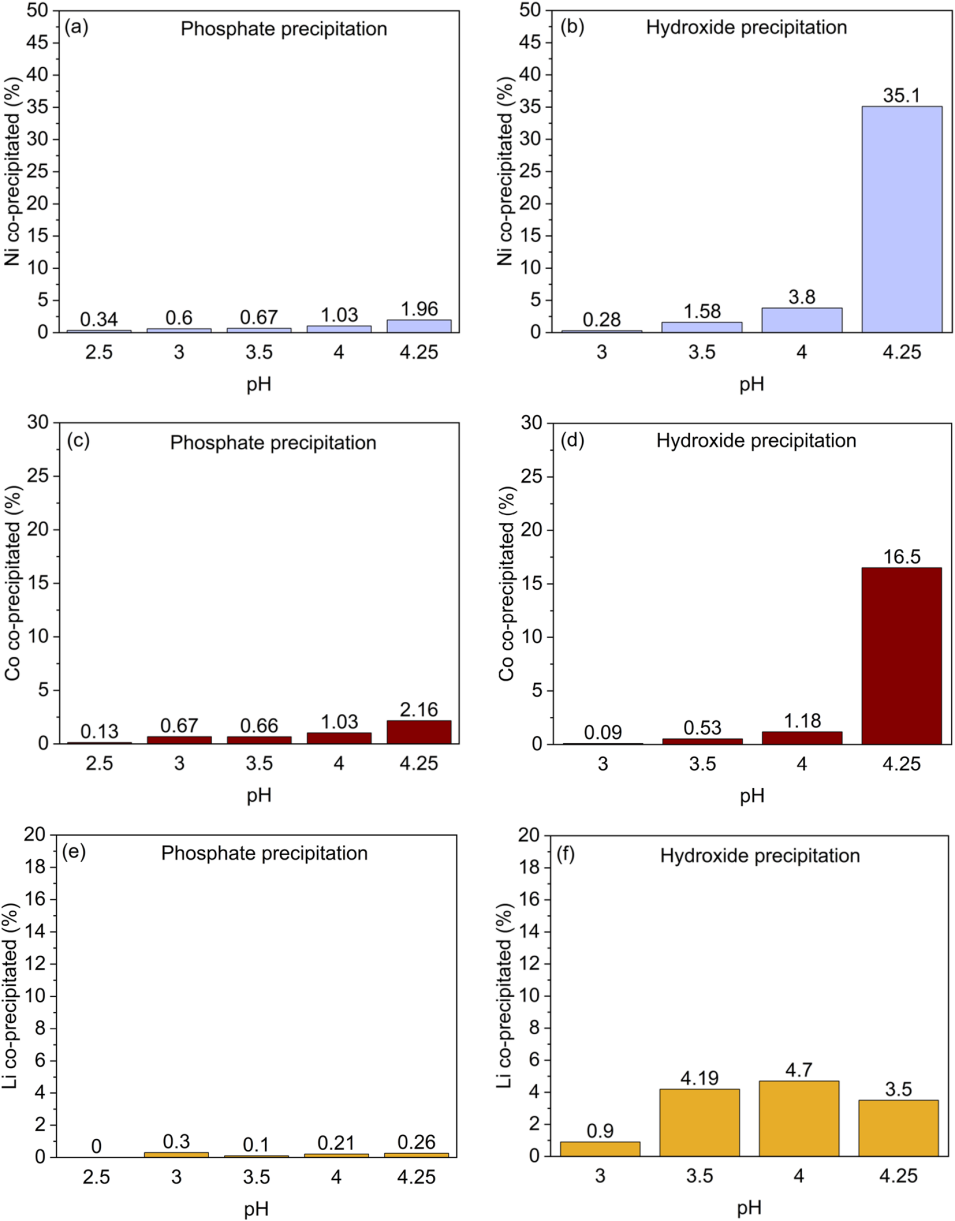
Co-precipitation of (**a**) Ni in phosphate precipitation (P1–P5) and (**b**) Ni in hydroxide precipitation (P6–P9), (**c**) Co in phosphate precipitation (P1–P5) and (**d**) Co in hydroxide precipitation (P6–P9), (**e**) Li in phosphate precipitation (P1–P5) and (**f**) Li in hydroxide precipitation (P6–P9) at t = 180 min, T = 60 °C, ω = 300 rpm.

Co-precipitation of Co, Ni, and Li was investigated in the phosphate and hydroxide systems. The behavior of nickel (Fig. 3a, b) and cobalt (Fig. 3c, d) followed a similar trend. In the phosphate system, co-precipitation of Ni and Co increased slightly with pH, both reaching ~ 2% at pH 4.25. In the hydroxide tests, co-precipitation of Ni and Co also increased slightly up to pH 4 (Ni ~ 4% and Co ~ 1%). After this, at pH 4.25, co-precipitation of Ni and Co increased dramatically to 35% and 17%, respectively. Lithium (Fig. 3e, f) co-precipitation was minor (≤ 0.3%) in phosphate precipitation at all investigated pH values, whereas in hydroxide precipitation it increased from 1 to > 4% as the pH was raised to 3.5 − 4.25. These results are in line with our previous work^11^. Additionally, valuable metal co-precipitation in the hydroxide process (pH 3.5 − 4.5) was found to be < 1% in the absence of aluminum^11,48^. The co-precipitation of metals could be caused by the absorption by aluminum hydroxide gel. Klaehn et al.^39^ found that in a phosphate process Fe and Al were precipitated completely at pH 4, while in hydroxide process only 24% of Al and 94% of Fe were precipitated along with ~ 11% of Ni and Co co-precipitated.

### Particle formation tracking

**Figure 4:**
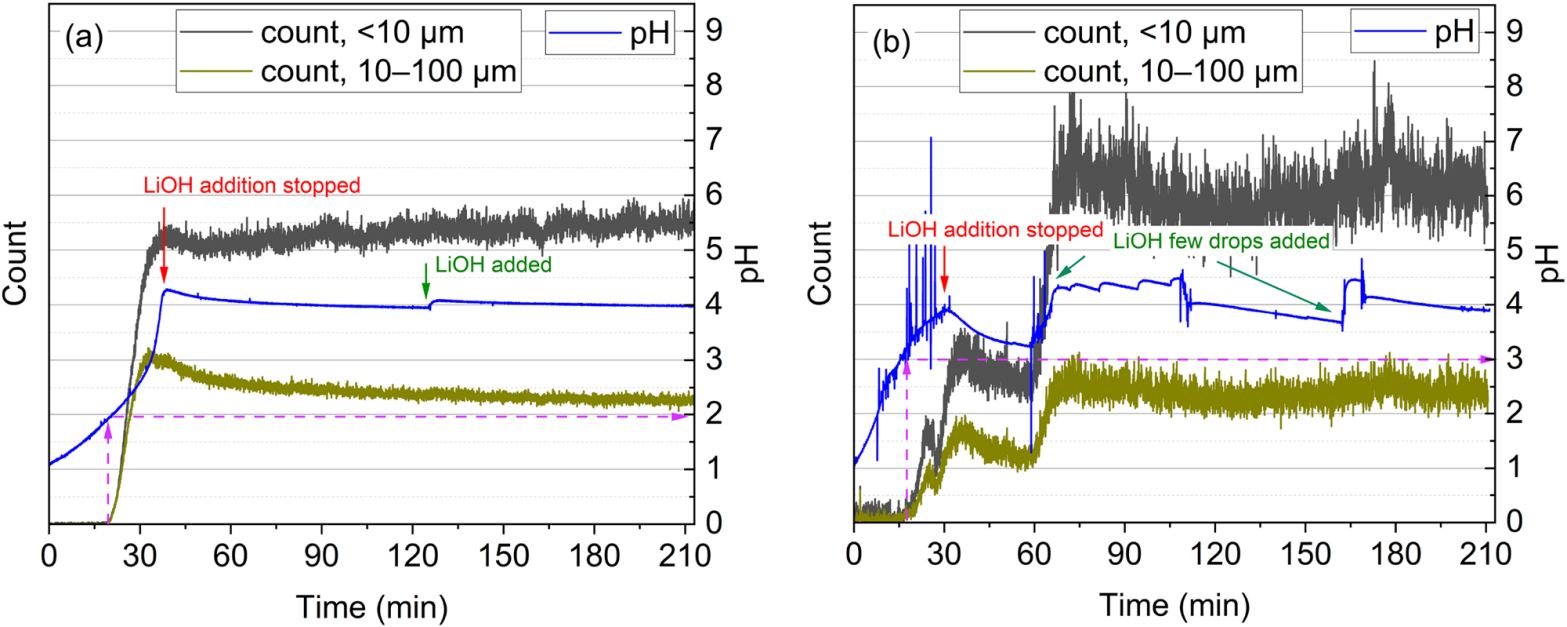
Particle count for (**a**) phosphate precipitation (test P4) and (**b**) hydroxide precipitation (test P8); pH = 4, t = 180 min, T = 60 °C, ω = 300 rpm.

The particle formation and pH were tracked throughout the tests in experiments P4 and P8. The particle tracking counts show that Fe-Al phosphate precipitation was initiated at a lower pH (pH 2) compared to hydroxide precipitation (pH 3), as shown in Fig. 4a, b. From the particle tracking it is evident that Fe and Al phosphate solids formed earlier, i.e., ~ 40 min after the start of the test, whereas the formation of hydroxide solids stabilized only after ~ 80 min, indicating the more favorable kinetics of the phosphate system in terms of reaching a constant particle count. The fast kinetics in phosphate precipitation were in line with the reaction time of 30 min of iron removal recorded by Masambi et al.^38^.

In phosphate precipitation (Fig. 4a), the pH and the amount of particles in the slurry were shown to remain relatively constant after the initial addition of LiOH was terminated. This indicates the completeness of Fe and Al crystallization at the selected pH by *t* = 40 min, with the minimum amount of consequent precipitation of other metals (as shown in Fig. 3). Figure 4b illustrates that, during the precipitation of hydroxide, the pH tended to fluctuate more in comparison to phosphate precipitation. In the case of hydroxide precipitation, the amount of solids in the slurry was seen to slightly decrease at around 30 min from the start of the test, between pH 3 and pH 3.25. Such a decrease may be attributed to the formation of iron oxide, its transformation to hydroxide, and the concurrent growth of aluminum hydroxide particles. In both the phosphate and hydroxide processes, an increase in < 10 µm particles during the process can be attributed to possible competing side reactions related to the hydrolysis of metal ions. It may indicate the partial precipitation of trivalent metals as hydroxides in the phosphate process. In the hydroxide process, however, the fluctuations in the < 10 µm particle count could be attributed to the slower kinetics of iron and aluminum precipitation as well as the co-precipitation of other metals, at least Ni, Co, and Li, as shown above in Fig. 3b, d, f.

### Filtration

**Figure 5:**
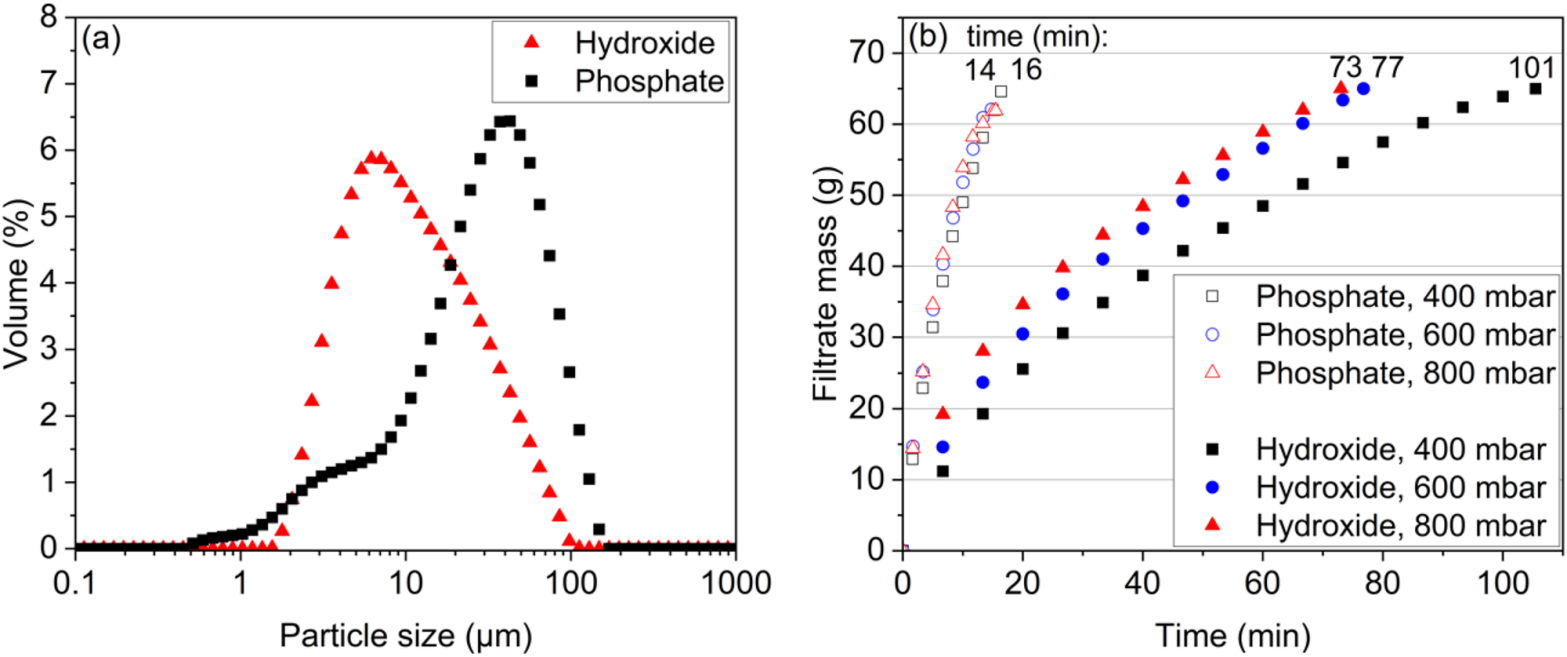
Particle size (**a**) and filtration rate (**b**) for slurries obtained in phosphate and hydroxide precipitation, tests P10FL and P11FL (pH = 4, t = 180 min, T = 60 °C, ω = 300 rpm).

The particle size distribution and the filtration rate of phosphate and hydroxide precipitation slurries obtained at pH 4 (P10FL and P11FL) were measured to compare the filterability of the precipitates. The average particle size of the phosphate cake was shown to be significantly larger than that of the hydroxide cake (Fig. 5a). Such a difference in particle size could partially explain the experimentally confirmed hypothesis in this work that the filtration rate of the phosphate (FePO_4_-AlPO_4_) cake was approximately 6 times higher than that of the hydroxide cake (Fe(OH)_3_-Al(OH)_3_), as shown in Fig. 5b. Earlier, Zhang et al.^27^ observed an improved filtration of the phosphate precipitate over hydroxide, whereas Masambi et al.^38^ also mentioned that iron phosphate was easy to filter. It has been suggested that the composition of the PLS has an impact on the filterability of hydroxide precipitate, i.e., the absence of aluminum improves the filtration performance^11^.

The compressibility factor (n) of the phosphate and hydroxide cakes was calculated using Eq. (22) and Eq. (23). The compressibility factor is close to 0 for incompressible cakes, 0.2–0.8 for most cakes, but can be over 1 for highly compressible cakes (Mineral Processing Design and Operations, 2016). The phosphate cake was found to be incompressible (n = 0.1), with the pore and particle properties remaining constant during filtration, thus maintaining a high filtration rate. Likewise, calcium carbonate has a slightly compressed cake (*n* = 0.19), according to Mahdi et al.^49^. The compressibility factor of the hydroxide cake, on the other hand, was found to be 0.86, indicating high compressibility, with amorphous particles being deformed, indicating the gel-like nature of the precipitate. The change in particle is suggested to result in a decrease in porosity and permeability, thus blocking the filter and affecting the filtration rate. The viscosity of both filtrates was measured (Fig. S3) to be the same and was used in the calculation of the compressibility factor. The XRD analysis (Fig. S4) confirmed that both the phosphate and hydroxide cakes were amorphous, which is in agreement with the literature^27,30,39^ as no matching compounds were found.

### Effect of fluoride

**Figure 6:**
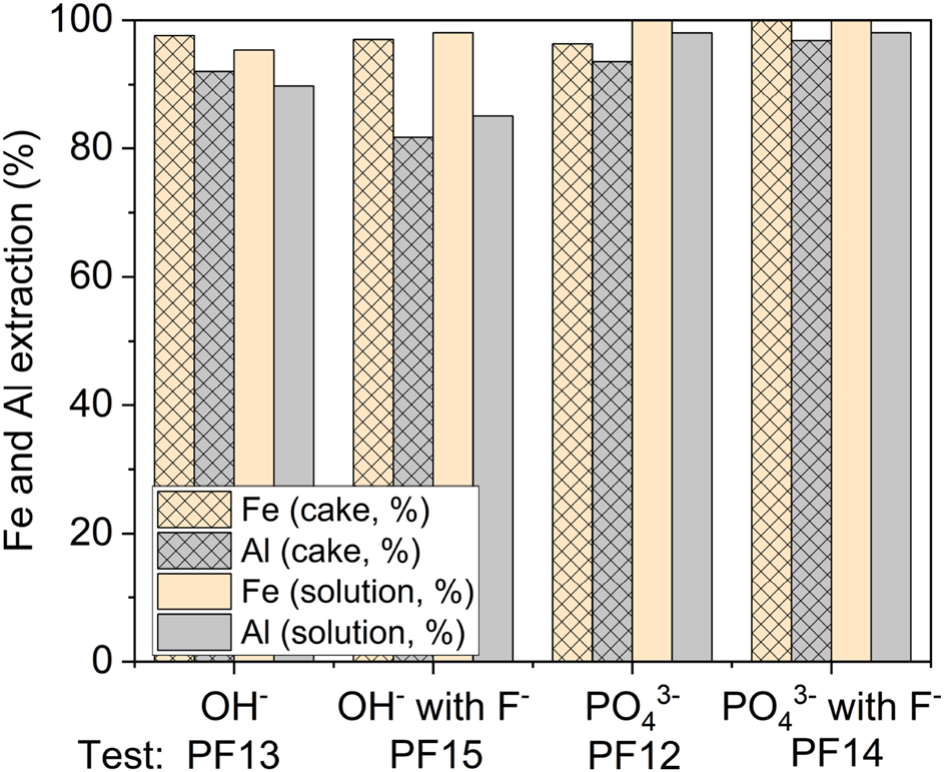
Precipitation of iron and aluminum as phosphate and as hydroxide, with (0.7 g/L) and without fluoride. Iron and aluminum extraction was calculated based on the cake (Eq. 20) and final solution (Eq. 21); (pH = 3.5, t = 180 min, T = 60 °C, ω = 350 rpm).

The effect of fluoride on precipitation performance was briefly investigated for the same synthetic leach solution with fluoride ions (0.7 g/L), PF12–PF15. The selected pH value of 3.5 (± 0.1) was maintained. As shown in Fig. 6, the iron and aluminum phosphate precipitation was near-complete unlike that of the hydroxide, which is in agreement with the main experimental series results (P3 and P7, Fig. 2). In the case of the phosphate precipitation process, iron recovery in the cake was complete (100%) with the presence of fluoride in the solution, while Al recovery was not affected. This is in agreement with the research of Jie et al.^30^, suggesting that the presence of fluoride in the solution could result in improved extraction of iron phosphate, as indicated by the results of tests PF12 and PF14 (Fig. 6). In the hydroxide precipitation process, the precipitation of aluminum decreased in the presence of fluorides (test PF15) as its extraction was 5–10% lower (test PF12). However, the recovery of iron was not affected by the presence of fluorides, suggesting that fluoride could interfere with aluminum hydroxide precipitation through the formation of strong Al-F complexes (Eqs. 10–14).

According to Table 5, which is based on elemental maps in supplementary material (Figs. S5–S8), fluoride remained in the solution possibly due to the formation of complexes with aluminum and interfered with its precipitation as fluoride was not observed in the precipitate (test PF15). In the case of phosphate process, fluoride was found in precipitate (PF14) indicating that the trivalent metals could have preferentially formed complexes with phosphate, hence some fluoride precipitated with remaining aluminum as aluminum fluoride.

**Table 5:** Qualitative elemental analysis results of precipitates obtained by EDS (wt.%).

Test	Conditions	Fe	Al	F	S	P
PF13	Hydroxide	11.27	19.05	–	7.98	0.26
PF15	Hydroxide + F	11.83	18.83	–	6.87	0.47
PF12	Phosphate	8.78	17.74	–	3.49	17.98
PF14	Phosphate + F	8.31	17.47	0.72	1.30	18.65

According to Jie et al.^30^ fluoride can be removed from solution with iron and aluminum in phosphate process. Ntuk et al.^50^ demonstrated that aluminum fluoride can readily precipitate at pH 5 in hydroxide process. Klaehn et al.^39^ found in their work, where Fe and Al phosphate and hydroxide precipitated from LIB leach solution at pH 4, the cakes contained ~ 3 wt.% fluoride. The positive effect of high temperature in aluminum hydroxide precipitation in the presence of fluoride was highlighted by Ntuk et al.^50^—the solubility of the main product (AlF_2_OH) was almost 1000 times lower at 90 °C compared to 25 °C in the hydroxide system.

Additionally, the co-precipitation of the other metals (Ni, Co, Mn, Li) in the solution (Table S4) was similar when compared to the main test series (Fig. 3), indicating that the co-precipitation behavior of these metals was not significantly affected by the presence of fluoride.

### Discussion

This research work demonstrates that the use of phosphate as precipitant enhances impurity removal (AlPO_4_ and FePO_4_) from hydrometallurgical LIB recycling processes. The moderate temperature of 60 °C was selected based on our previous work experience and literature. Higher temperatures can affect the precipitation kinetics and pH, and thus improve Al precipitation. The technological feasibility of phosphate precipitation looks promising, with low co-precipitation of valuable battery elements (< 2% at pH 4). With the aim of developing sustainable recycling processes, not only technological process performance but also environmental competitiveness is of high importance^26^. Although process modeling and life cycle analysis were beyond the scope of the current investigation, the carbon footprint of the precipitation chemicals can be briefly compared. The global warming potential (GWP) of producing one kilogram of H_3_PO_4_ is 1.41 CO_2_-eq/kg (ReCiPe 2016 midpoint method), while the GWP of the neutralization chemical LiOH used in its production is much higher than that of NaOH (5.86 vs. 1.22 CO_2_-eq/kg, ReCiPe 2016 midpoint method). While LiOH might not be a more economically feasible agent than e.g. NaOH, however, in the current study, LiOH was selected to avoid accumulation of Na^+^ impurity in the process and to prove its efficacy as precipitating agent. Inspired by the outcomes of this work, LiOH alone or mixed with NaOH could be considered to avoid the introduction of a separate unit for Na^+^ removal later in the process. The results show that the phosphate precipitation process has 5% lower consumption of LiOH compared to the studied hydroxide process where only LiOH was used. Based on the difference in actual consumption of chemicals (LiOH and H_3_PO_4_) at pH 4 in the current laboratory-scale study, the GWP of the chemicals required (solely for Fe and Al removal) suggests slightly higher values for the phosphate process (135 CO_2_-eq/m^3^) in comparison to the hydroxide process (117 CO_2_-eq/m^3^). However, despite the lower GWP of the chemical use in this single precipitation process, the challenges and consequent impact on the GWP of the holistic process—in the hydroxide process arise from the valuable metal loss in co-precipitation, longer reaction time, as well as the slowness of the filtration process. Alternative phosphate sources as well as low acid leaching^51^ could potentially decrease the need for virgin chemicals. Nevertheless, it is important to consider upscaling such process in order to study the performance on an industrial scale.

This research work demonstrates that phosphate precipitation can provide a versatile and efficient strategy for the purification of hydrometallurgical Li-battery leach solution. Further, phosphates could potentially be used to recover lithium as Li_3_PO_4_ at a basic pH value^2,48,52,53^. Additionally, the amount of emerging LFP batteries is expected to increase significantly in the coming years^54–56^. Such an increase in LFP chemistry would introduce new types of waste streams with a large amount of phosphate containing black mass into hydrometallurgical LiB recycling. Furthermore, this would potentially increase the feasibility of recovering Al, Fe, and even Li as phosphates by providing an in-situ precipitation agent in the process and minimizing the need for hydroxide chemicals required for aluminum and iron removal as well as improving the filterability of the solids.

In the studied system, the co-precipitation of fluoride at low pH (e.g., 4) is not detrimental to the system as it can be fully recovered along with Fe and Al and treated separately; however, it may affect the Al removal from the solution. The current results provide only an indicative interpretation of fluoride incorporation in phosphate and hydroxide systems, and therefore fluoride-containing solution purification needs to be investigated further to fully understand the distribution of fluoride and its impact on different process streams. Further, in the spirit of the circular economy, more ambitious targets for low value element (Al, Fe, F) recovery as products need to be addressed in future recycling processes.

## Conclusions

We investigated the removal of trivalent iron and aluminum from synthetic Li-battery leach solution as phosphates and hydroxides. The novel results demonstrate that the use of phosphoric acid in phosphate precipitation was found to be more efficient than the more conventional solution purification by hydroxide precipitation. Phosphate precipitation was characterized by a rapid reaction between the phosphate and trivalent metal ions, with less than 2% of the valuable battery metals (Li, Co, Ni) incorporated in the phosphate cake. In hydroxide precipitation, over 4% of Li, Co, and Ni were lost in the process. Co-precipitation of Co and Ni increased with increasing pH in both the phosphate and hydroxide processes, whereas, in the hydroxide process, Li loss remained the highest in the pH range of 3.5–4. With the novel method applied in this work to track particle formation, it was found that the particle growth initiation and precipitation were completed at a lower pH in phosphate precipitation than in hydroxide precipitation. The filtration rate and compressibility index, as the key cake properties relevant to an industrial-scale hydrometallurgical process, were also evaluated. This novel finding demonstrates the the superiority of the phosphate cake in terms of filterability over hydroxide: the phosphate cake filtration rate was 6 times higher than that of the hydroxide cake and its compressibility index was calculated to be 0.1, thus incompressible. The compressibility index of the hydroxide cake was 0.86, which is highly compressible and suggests that it would be highly challenging in the filtration process. XRD analysis confirmed the amorphous nature of cakes obtained in both the phosphate and hydroxide processes. The results demonstrate that the phosphate precipitation of Fe and Al is not only relevant for NMC and LCO type battery recycling but may also in future be applied for the synergistic leaching of the emerging LFP battery waste fraction.

The short study on fluoride behavior demonstrated a potentially negative effect on aluminum extraction in hydroxide precipitation, as aluminum recovery was 5–10% lower with fluorides at pH 3.5. We suggest that this is due to the formation of Al-F complexes in solution, making aluminum more stable in dissolved form. During phosphate precipitation, fluoride was found to have no clear effect on phosphate precipitation. Nevertheless, the behavior of the system with fluoride present during crystallization should be investigated in more detail to assess the possibility of complete fluoride removal from the solution together with impurity metals (Al and Fe) in the range of acidic pH values. Successful co-precipitation of fluoride concurrently with impurity metals could decrease its accumulation in subsequent process steps and streams, such as solvent extraction streams.

### Supplementary Information

Supplementary Information.

## Data Availability

The datasets used and/or analysed during the current study available from the corresponding author on reasonable request.
