# Supplementary material for: Fe3+ and Al3+ removal by phosphate and hydroxide precipitation from synthetic NMC Li-ion battery leach solution
Source: Sci Rep. 2023 Dec 5;13:21445. doi: 10.1038/s41598-023-48247-6 (PMC10698013; doi:10.1038/s41598-023-48247-6)
Supplement: Supplementary file 1 — Supplementary Information. [file 41598_2023_48247_MOESM1_ESM.docx]

# Supplementary material

Fe^3+^ and Al^3+^ removal by phosphate and hydroxide precipitation from Li-ion battery leach solution

Alexander Chernyaev^*^, Jianxin Zhang, Sipi Seisko, Marjatta Louhi-Kultanen, Mari Lundström

Aalto University, School of Chemical Engineering, Department of Chemical and Metallurgical Engineering, 00076 Aalto, Finland

*Corresponding author: [alexander.chernyaeav@aalto.fi](mailto:alexander.chernyaeav@aalto.fi)

## Lithium hydroxide consumption


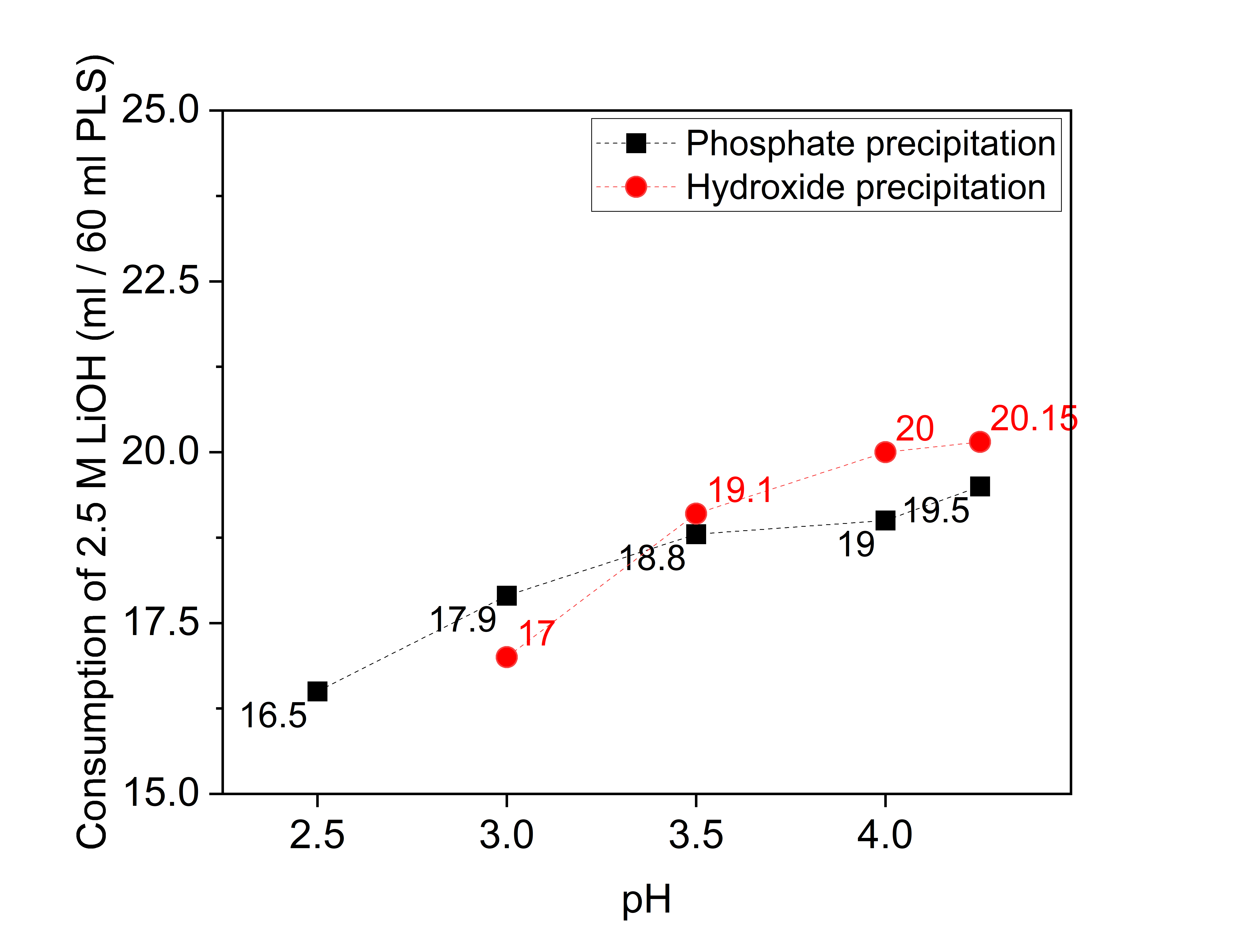


Fig. S1. Total consumption of 2.5 M LiOH solution in tests P1-P9.

## Metal concentration in initial and final solution

Table S1. Metal concentration in initial solution, final and in wash water (both latter in brackets), mg/L.

| Exp. | pH | Li | Al | Fe | Ni | Co | P* |
| --- | --- | --- | --- | --- | --- | --- | --- |
| P1 | 2.5 | 4100 (6927, 776) | 4332 (1615, 236) | 1814 (57.7, 7.8) | 11064 (9618, 1099 | 4975 (4332, 495) | 5100 (1305, 186) |
| P2 | 3 | 4123 (7095, 964) | 4300 (3.9, 2) | 1854 (6, 0) | 11594 (8498, 1142) | 5355 (3880, 549) | 5100 (15.6, 1) |
| P3 | 3.5 | 4101 (6275, 445) | 4418 (1.24, 0.5) | 1867 (25, 1) | 11475 (7439, 536) | 5318 (3501, 268) | 5100 (2.3, 0) |
| P4 | 4 | 4110 (7422, 1284) | 4310 (3.4, 1) | 1866 (0, 0) | 11288 (8400, 1449) | 5599 (4087, 718) | 5100 (20.4, 4.76) |
| P5 | 4.25 | 4100 (7076, 759) | 4330 (1.31, 0.15) | 1810 (0.17, 0.01) | 11060 (8565, 933) | 4977 (3834, 427) | 5100 (19, 3.3) |
| P6 | 3 | 4100 (6779, 829) | 4332 (3121, 402) | 1814 (288, 9.57) | 11064 (11453, 1387) | 4975 (5162, 261) |  |
| P7 | 3.5 | 4110 (7019, 1396) | 4310 (183, 38.82) | 1866 (3, 0) | 11288 (8552, 1627) | 5599 (4318, 839) |  |
| P8 | 4 | 4101 (6146, 560) | 4300 (6, 2) | 1867 (7, 0) | 11475 (7451, 656) | 5318 (6307, 327) |  |
| P9 | 4.25 | 4186 (7614, 1434) | 4500 (0, 0) | 1715 (0, 0) | 11330 (5207, 961) | 5354 (3480, 644) |  |
| PF12 | 3.5 | 4100 (11464, 1816) | 4332 (92.9, 27.7) | 1814 (2.43, 0.31) | 11064 (14000, 2253) | 4975 (6277, 1012) |  |
| PF13 | 3.5 | 4100 (5940, 501) | 4332 (244, 78) | 1814 (67.7, 3.8) | 11064 (8101, 670) | 4975 (3787, 315) |  |
| PF14 | 3.5 | 4075 (7487, 788) | 4277 (53, 19.6) | 1875 (0.32, 0.22) | 10462 (8682, 921) | 4870 (4035, 432.7) |  |
| PF15 | 3.5 | 4075 (6983, 424) | 4277 (552, 37) | 1875 (32.24, 1.63) | 10462 (8883, 515) | 4870 (4196, 244) |  |

*Calculated initial concentration of P based on the amount of added 85% H_3_PO_4_ (0.65 ml) to the initial solution

## Filterability tests

Table S2. Measured constant filtration properties used in cake compressibility calculation

| Parameter | Hydroxide cake | Phosphate cake | Unit |
| --- | --- | --- | --- |
| $c$ | 20.04 | 35.3 | kg/m^3^ |
| $\mu_{l}$ | 0.02 | 0.02 | Pa s |
| $A$ | 0.00196 | 0.00196 | m^2^ |


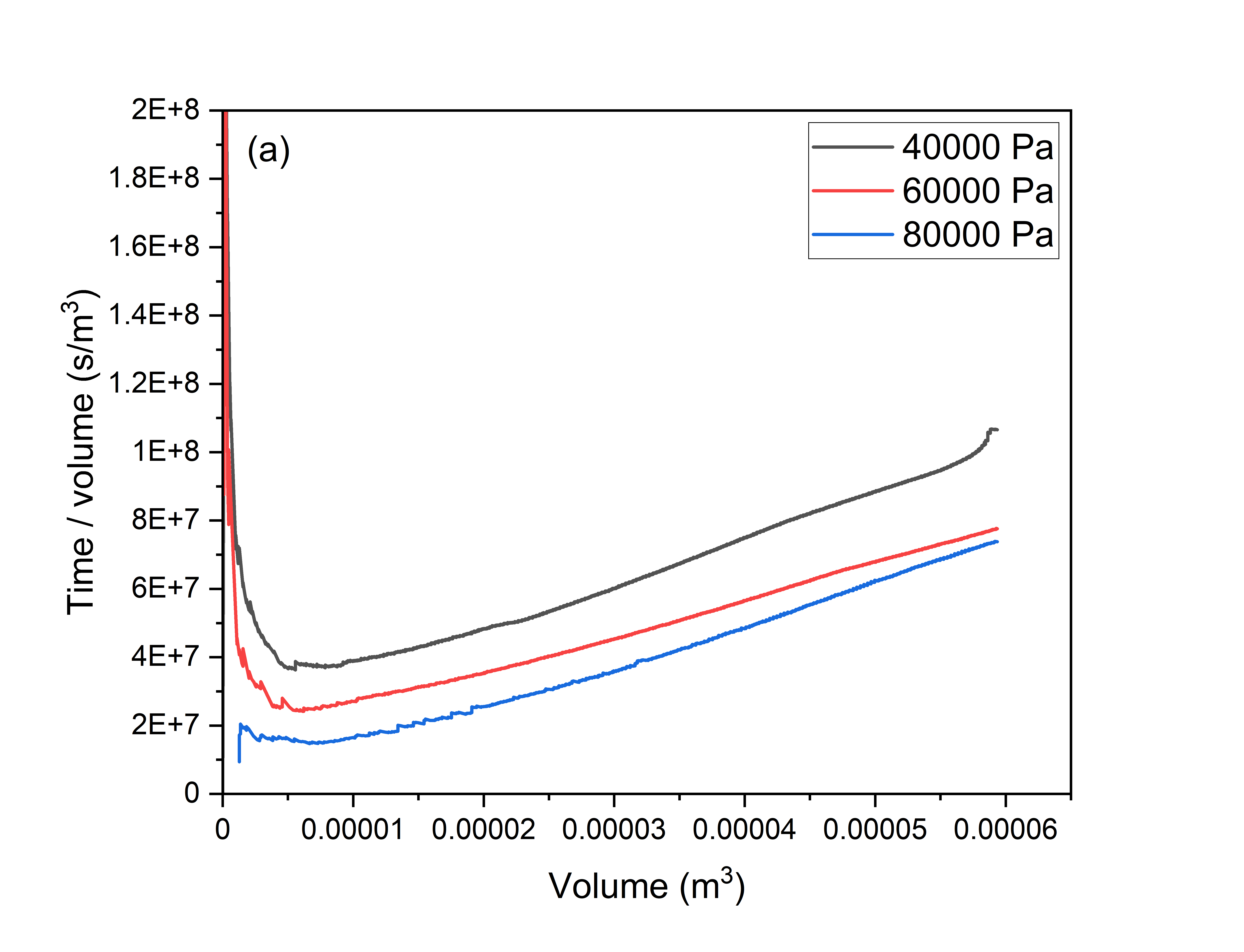

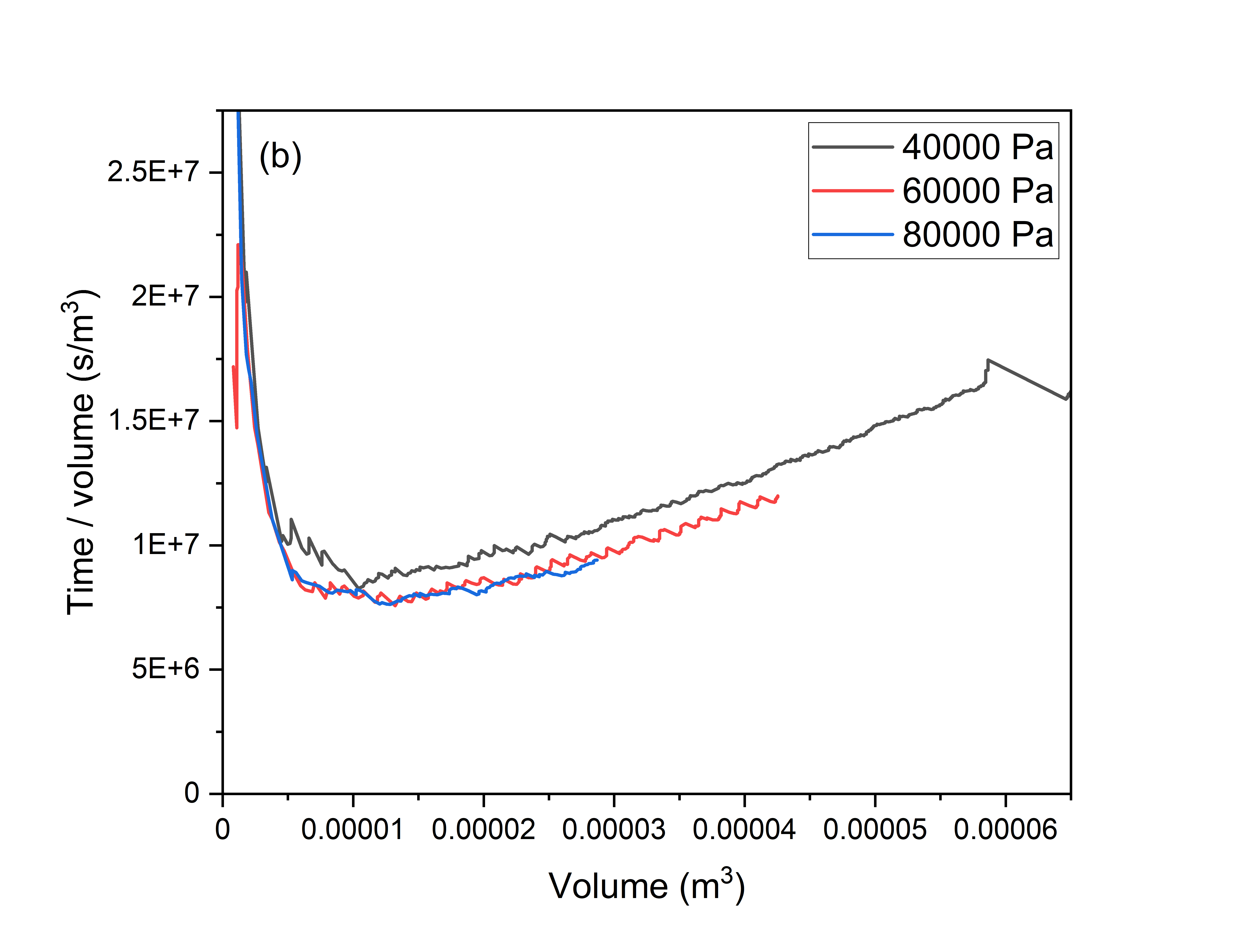


Fig. S2. Filtration data plotted as time/volume vs. volume for (a) hydroxide cake and (b) phosphate cake used in average cake resistance calculation (tests P4 and P8, pH = 4, t = 180 min, T = 60 °C, ω = 300 rpm).

Table S3. Calculated specific resistance, $\alpha_{av}$ (m/kg), used in compressibility index calculation

| Pressure, Pa | $\alpha_{\mathrm{av}}$  Hydroxide cake | $\alpha_{\mathrm{av}}$  Phosphate cake |
| --- | --- | --- |
| 40000 | 4.23×10^8^ | 3.27×10^7^ |
| 60000 | 5.00×10^8^ | 3.90×10^7^ |
| 80000 | 7.82×10^8^ | 3.45×10^7^ |


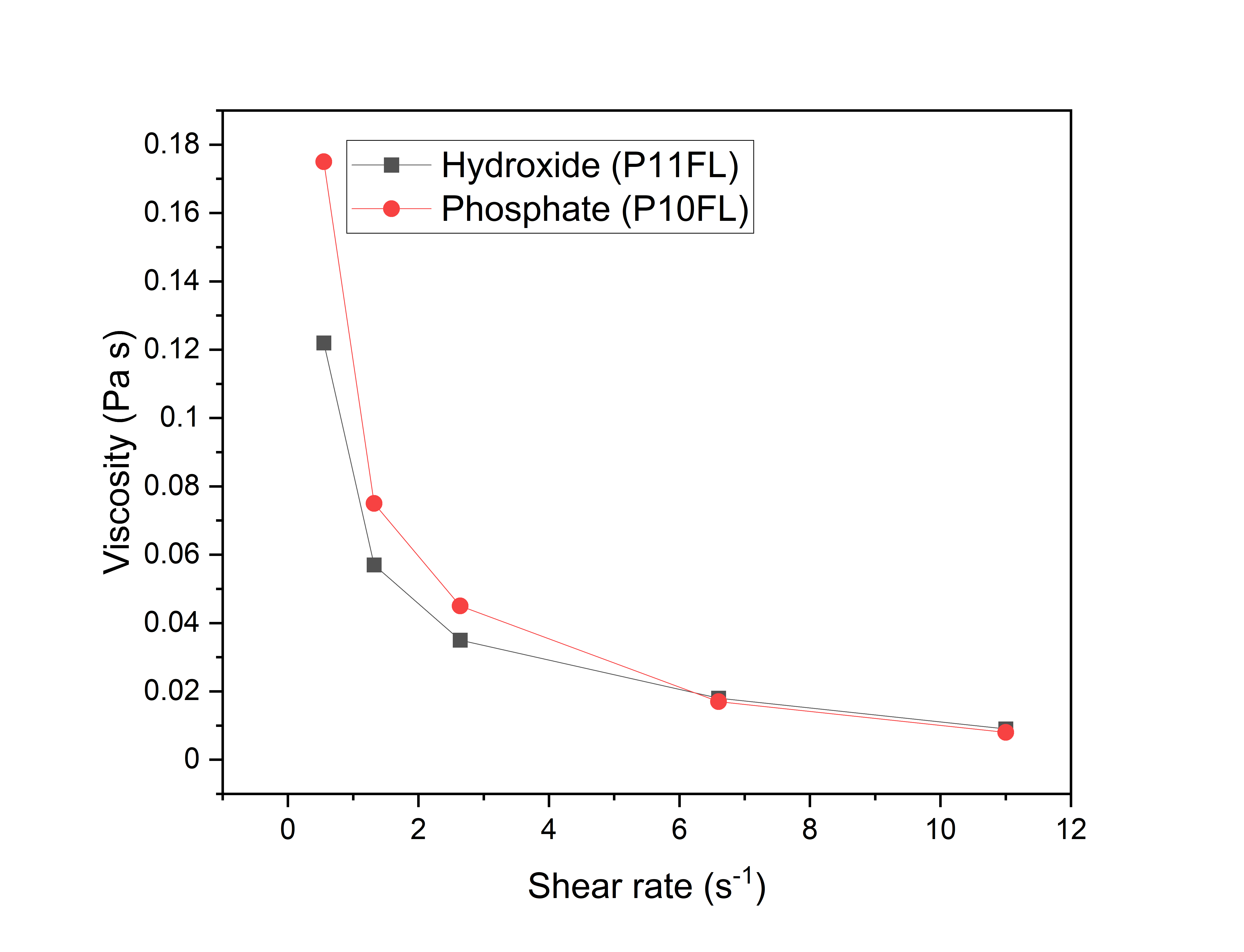


Fig. S3. Viscosity of filtrates obtained in filtration test.

## Precipitation in fluoride containing solutions

Table S4. Co-precipitation of metals (%)

| Test | Ni | Mn | Co | Li |
| --- | --- | --- | --- | --- |
| Hydroxide (PF13) | 1.53 | 0.45 | 0.51 | 4.44 |
| Phosphate (PF12) | 1.68 | 0.24 | 0.58 | 0.18 |
| Hydroxide with fluoride (PF15) | 1.74 | 0.44 | 0.55 | 3.76 |
| Phosphate with fluoride (PF14) | 0.80 | 0.99 | 0.65 | 0.10 |

## XRD


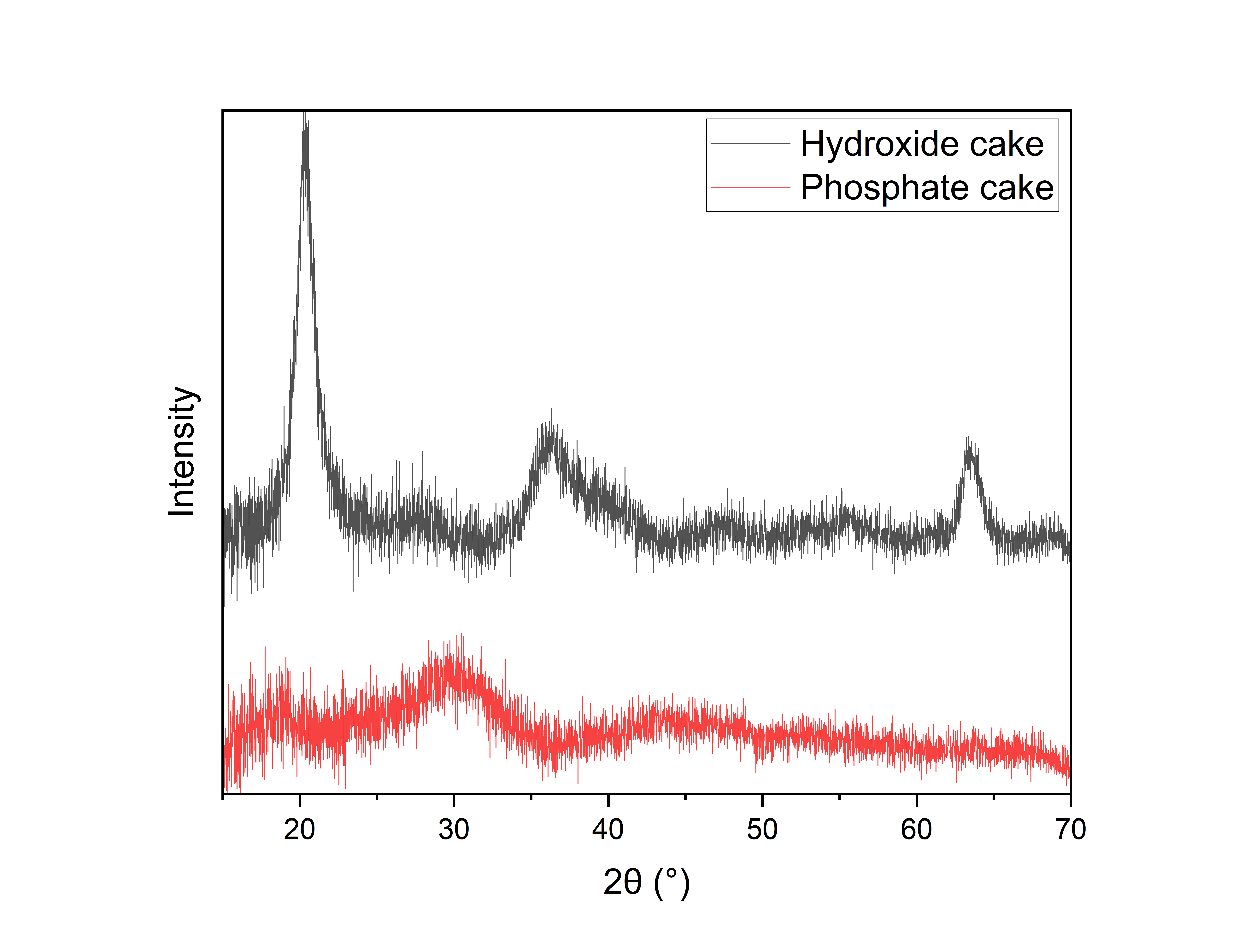


Fig. S4. XRD diffractogram of cakes obtained from tests P4 and P10.

## EDS elemental mapping


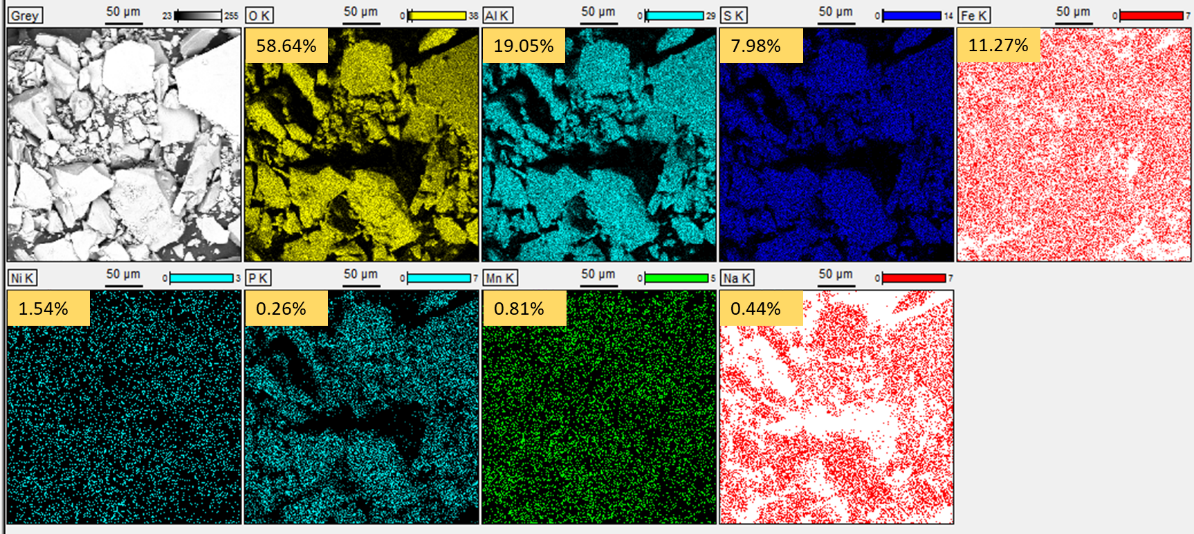


Fig. S5. Elemental map of precipitate obtained in test PF13.


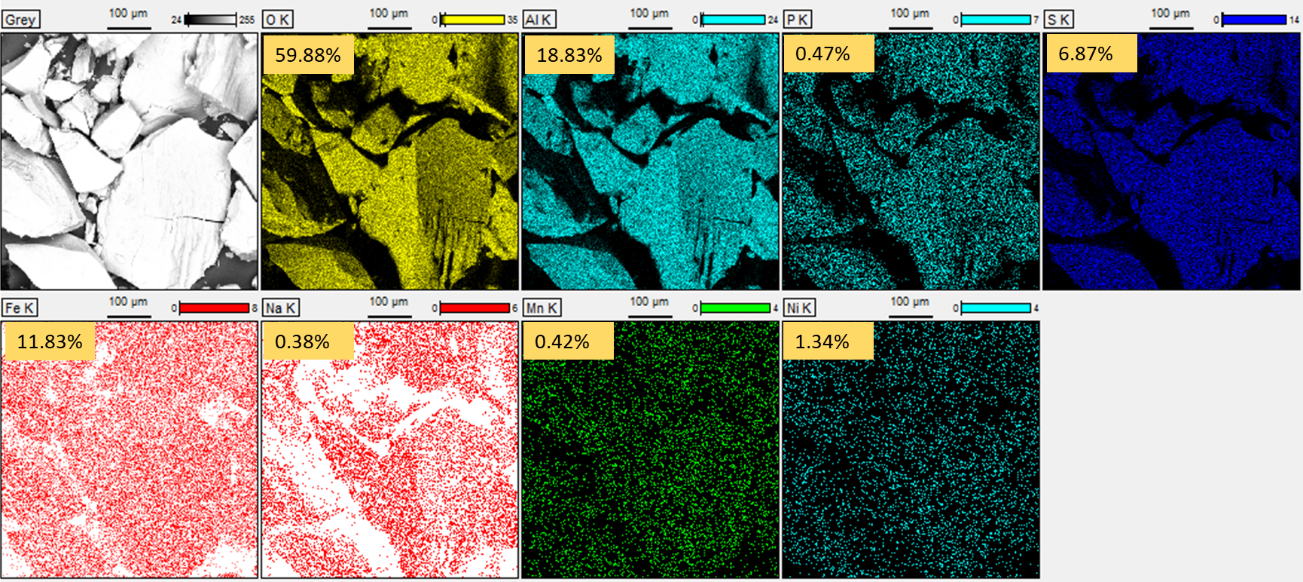


Fig. S6. Elemental map of precipitate obtained in test PF15.


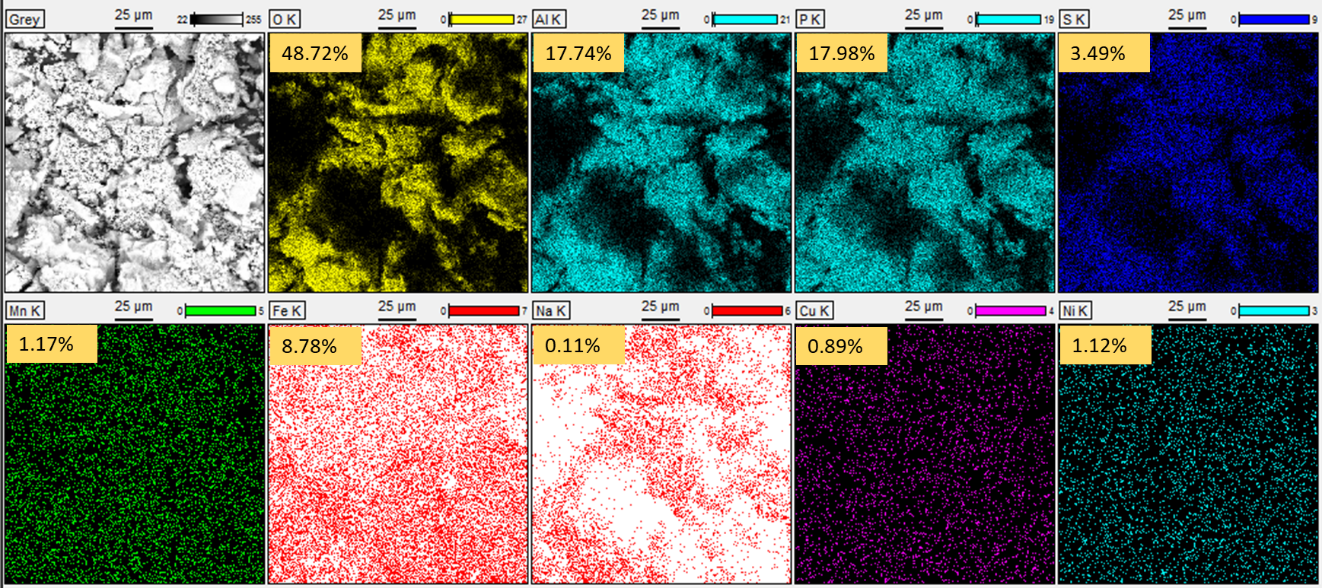


Fig. S7. Elemental map of precipitate obtained in test PF12.


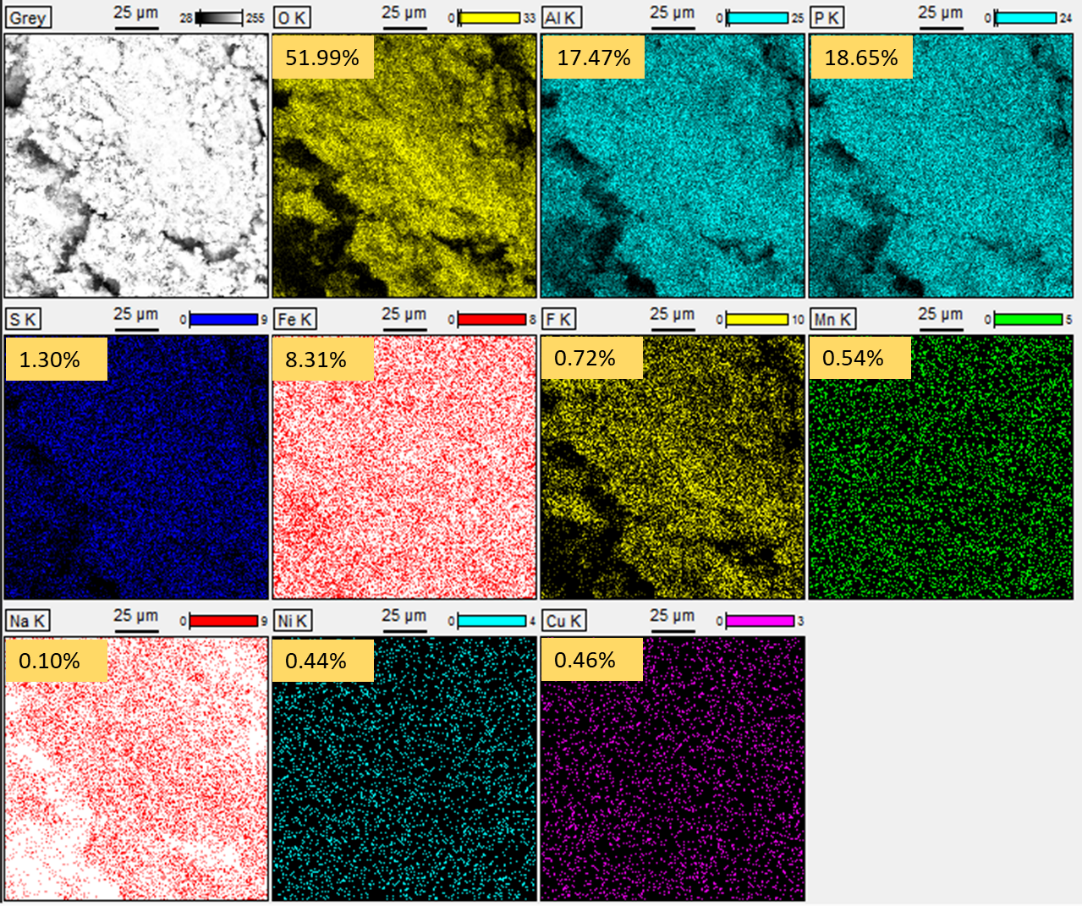


Fig. S8. Elemental map of precipitate obtained in test PF14.
